# Potential Epigenetic Mechanism in Non-Alcoholic Fatty Liver Disease

**DOI:** 10.3390/ijms16035161

**Published:** 2015-03-05

**Authors:** Chao Sun, Jian-Gao Fan, Liang Qiao

**Affiliations:** 1 Department of Gastroenterology, Xinhua Hospital, Shanghai Jiaotong University School of Medicine, Shanghai 200092, China; E-Mail: csun7682@163.com; 2 Storr Liver Centre, Westmead Millennium Institute for Medical Research, University of Sydney, the Westmead Clinical School, Westmead Hospital, Westmead, NSW 2145, Australia

**Keywords:** epigenetics, non-alcoholic fatty liver disease (NAFLD), non-alcoholic steatohepatitis (NASH), DNA methylation, histone modifications, microRNA

## Abstract

Non-alcoholic fatty liver disease (NAFLD) is characterized by excessive fat accumulation in the liver. It ranges from simple steatosis to its more aggressive form, non-alcoholic steatohepatitis (NASH), which may develop into hepatic fibrosis, cirrhosis, or hepatocellular carcinoma (HCC) if it persists for a long time. However, the exact pathogenesis of NAFLD and the related metabolic disorders remain unclear. Epigenetic changes are stable alterations that take place at the transcriptional level without altering the underlying DNA sequence. DNA methylation, histone modifications and microRNA are among the most common forms of epigenetic modification. Epigenetic alterations are involved in the regulation of hepatic lipid metabolism, insulin resistance, mitochondrial damage, oxidative stress response, and the release of inflammatory cytokines, all of which have been implicated in the development and progression of NAFLD. This review summarizes the current advances in the potential epigenetic mechanism of NAFLD. Elucidation of epigenetic factors may facilitate the identification of early diagnositic biomarkers and development of therapeutic strategies for NAFLD.

## 1. Introduction

Nonalcoholic fatty liver disease (NAFLD) is a common and complex liver disease, with the risk factors including central obesity, dyslipidemia, hypertension, insulin resistance (IR), and type 2 diabetes mellitus (T2DM) [1,2]. Patients with NAFLD may develop non-alcoholic steatohepatitis (NASH), which is defined as hepatic steatosis and inflammation or liver cell injury, with or without fibrosis [3,4]. NASH may progresss to fibrosis or cirrhosis [5], leading to hepatocellular carcinoma (HCC) [6] and even liver-related mortality [7]. Due to unhealthy lifestyles and dietary patterns, the population of obese individuals has increased globally over the past few decades. NAFLD has become the most common cause of liver disorders worldwide [8]. In China, the reported prevalence of NAFLD is about 15% among the adults in the mainland and 27.3% in Hong Kong adults [8,9,10], but the proportion of NAFLD patients with liver fibrosis is low (3.7%) [11]. Several studies have shown that 26%–37% of patients with NASH will progress to hepatic fibrosis and 9% to cirrhosis over the next six years [12,13,14]. Based on long term follow-up studies, the prevalence of HCC is 0%–0.5% in NAFLD patients and 0%–2.8% in NASH patients [15,16].

The pathogenesis of NAFLD is multi-faceted and complicated. The liver plays a major role in lipid and glucose metabolism and NAFLD represents impaired homeostasis of the metabolism [17]. In the well-recognized double-hit theory of the pathogenesis of NAFLD, the first hit is the accumulation of triglycerides (TG) in hepatocytes, followed by a second hit in which inflammatory mediators cause hepatocellular injury, inflammation, and fibrosis [18,19,20]. Studies performed over the past few years have described a new model in which multiple parallel hits might be responsible for the development of NAFLD. Fatty acids and the metabolites facilitate the development of simple steatosis (SS) to NASH. IR enhances the recruitment of free fatty acids from the circulation and hepatic deposition of free acids, which activates endoplasmic reticulum (ER) and oxidative stress and ultimately apoptosis of hepatocytes. Fatty acids also enhance hepatic IR, which induces a vicious cycle of lipid storage [21,22].

Generally, NAFLD is caused by interactions between many environmental and genetic factors. However, the precise pathogenesis of NAFLD is not well understood. NAFLD is associated with changes at the transcriptional level that influences gene expression and phenotype [23]. Although most epigenetic aberrations are transient and non-heritable, some are transgenerational [24,25,26]. The most thoroughly studied markers for epigenetic alterations are DNA methylation, histone modifications, and the actions of microRNAs (miRs) [23]. These processes are regulated by the environmental factors such as nutrition and diet [27], drugs [28], and stress [29]. Epigenetic modifiers are involved in lipid metabolism, IR and ER stress, mitochondrial damage, oxidative stress, and inflammation. This can induce hepatic lipid accumulation and eventually NAFLD [30,31,32,33]. Epigenetic dysregulation has been found to trigger carcinogenesis of hepatocytes and facilitate the progression of HCC [34,35]. Evaluating epigenetic changes and their roles in the pathnogenesis of NAFLD would guide us to develop novel approaches for the prevention or therapy for the metabolic liver diseases.

## 2. DNA Methylation

Aberrant DNA methylation represents one of the major epigenetic changes that contribute to abnormal gene expression in the pathogenesis of NAFLD [36]. DNA methylation is catalyzed by DNA methyltransferases (DNMTs), which convert cytosine to 5-methylcytosine binding with guanine in DNA [37]. DNA methylation is regarded as a key process involved in changing the liver phenotype from normal liver through SS to NASH [38]. Usually, hypermethylation of CpG islands in gene promoters induces gene silencing and inhibits gene expression, and hypermethylation of the global DNA influences genomic stability [39]. It is essential to understand the role of DNA methylation in nutrition and genetics.

Diet is one of the main elements that influence DNA methylation. DNA methylation relies on the availability of *S*-adenosylmethionine (SAM), and the methyl donors from foods (including folate, betaine, and choline) are associated with SAM synthesis [40,41]. Folate is a catalytic substrate for transfering one-carbon units. Dysregulation of single-carbon metabolism may contribute to the hepatic steatosis by DNA methylation reaction. Folate deficiency has been proved to induce TG accumulation in the liver [42]. It has been reported that folate affects the expression of genes involved in fatty acid synthesis [43]. Another important methyl donor is betaine, which has been found to alleviate high-fat-diet-induced (HFD-induced) fatty livers [44]. Betaine supplementation has been showed to be associated with significantly less methylation of the microsomal triglyceride transfer protein (*MTTP*) promoter and more methylation of the genome. This promotes hepatic TG export and attenuates liver steatosis in NAFLD [38]. High-fat-sucrose-induced (HFS-induced) liver fat accumulation was reverted by the supplementation with methyl donors containing folic acid, choline, betaine, and Vitamin B12, which decreased the liver global DNA methylation and changed the methylation levels of CpG sites in the sterol regulatory element binding transcription factor 2 (*Srebf2*), 1-acylglycerol-3-phosphate Oacyltransferase 3 (*Agpat3*), and estrogen receptor 1 (*Esr1*) promoter regions [45]. Similarly, the methyl donor supplementation mediated fatty acid synthase (*FASN*) DNA hypermethylation, which may be involved in the improvement of HFS-induced NAFLD [46]. Methyl-deficient diets were found to reduce the concentration of hepatic SAM and resulted in CpG island methylation of 164 genes in mouse livers. These genes were found to be involved in DNA damage and repair, lipid and glucose metabolism, and the progression of fibrosis [47].

DNA methylation can be inherited from parents and passed to the next generation through markings on the chromosome [48]. It has been reported that maternal HFD can result in hepatic dysfunction in offspring during early postnatal life [49]. Cyclin-dependent kinase inhibitor 1A (*CDKN1a*), an inhibitor of the hepatic cell cycle, was hypomethylated at first exon and CpG dinucleotides in maternal HFD offspring, and the upregulated expression of *CDKN1a* was found to be correlated with hepatocyte growth in pathological states [49]. A similar study showed maternal Western diet during prenatal and post-weaning periods to increase the susceptibility of male offspring to NAFLD [50]. It has recently been shown that melatonin can reverse the methylation of leptin and ameliorate glucocorticoid-induced hepatic steatosis [51].

Peroxisome proliferator-activated receptors (*PPARs*) play a key role in adipogenesis and contribute to liver steatosis [52]. The balance of *PPARα* and *PPARγ* activity is associated with the synthesis of fatty acids and oxidation. *PPARα* modulates the activity of the proteins involved in protein transport and liver cytosolic fatty acid-binding activity (e.g., fatty acid translocase). Results have shown that the expression of *PPARα* is downregulated in liver steatosis [53], thereby favoring lipogenesis during oxidation. However, *PPARγ* regulates differentiation and cytokine production and has been found to reduce the expression of pro-inflammatory cytokines. One study reported that folic acid supplements could downregulate the methylation of *PPARγ* promoter and therefore upregulate the expression of *PPARγ* [54]. In addition, epigenetic modifications of *PPARγ* in the liver of NAFLD patients contribute to IR. Methylation levels of liver *PPARγ* coactivator 1α (*PGC1α*) and mitochondrial transcription factor A (*TFAM*) were relevant to fasting insulin and homeostasis model assessment of IR (HOMA-IR). Also, the hepatic level of mitochondrial DNA (mtDNA) is much higher in normal livers than in the NAFLD livers and was found to be inversely associated with PGC1α methylation, fasting insulin, and HOMA-IR [55].

Accumulating amounts of evidence have suggested that epigenetic variations of mtDNA methylation may occur during the development of NAFLD [56,57]. Mitochondria are the major sources and targets of reactive oxygen species (ROS). Oxidative stress can result in apoptosis due to impaired proton translocation, electron transport, and ATP synthesis [58]. The mitochondrially encoded NADH dehydrogenase 6 (*MT-ND6*) gene is a target of mitochondria methylation in NAFLD [32]. It has been reported that *MT-ND6* is highly methylated and that there is considerably less expression of *MT-ND6* mRNA in NASH patients than in SS patients. In addition, liver methylation of *MT-ND6* was found to be related to the severity of NAFLD indicating that the epigenetic modification of mitochondrial gene plays a critical role in the development and pathogenesis of NAFLD [32].

These data, which were collected in animal models of NAFLD, have also been demonstrated in human NAFLD patients. Murphy and colleagues have found 69,247 methylated CpG sites in NAFLD patients [59]. Methylation of these genes is involved in tissue repair and metabolic regulation [59]. Another study reported changes in the methylation of nine NAFLD-related genes coding for critical enzymes in intermediate metabolism and insulin-like signaling [60]. These methylations can be partially reversed after bariatric surgery [60]. Collectively, methylation modification with gene expression profiles is a powerful approach to identifying the pathophysiological pathways related to the development of NAFLD (Table 1).

**Table 1:** Target genes related to DNA methylations and histone modifications in NAFLD.

Mechanism	Study Subject	Target Gene(s)	References
DNA methylation	Mouse	*MTTP*	Wang *et al.* [38]
	Rat	*Srebf2*, *Agpat3*, *Esr1*	Cordero *et al.* [45]
	Rat	*FASN*	Cordero *et al.* [46]
	Rat	*CDKN1a*	Dudley *et al.* [49]
	Mouse	*PPARα*, *INSIG*, and *FASN*	Pruis *et al.* [50]
	Rat	*Leptin*	Tiao *et al.* [51]
	Rat	*PPARγ*	Sie *et al.* [54]
	Human	*PGC1α*, *TFAM*	Sookoian *et al.* [55]
	Human	*MT-ND6*	Pirola *et al.* [32]
	Human	*PC*, *ACLY*, *PLCG1*, *IGF1*, *IGFBP2*, *PRKCE*, *GALNTL4*, *GRID1*, *IP6K3*	Ahrens *et al.* [60]
Histone modificaitions	Macaques	*GPT2*, *DNAJA2*, *Rdh12*, *Npas2*	Aagaard-Tillery *et al.* [61]
	Mouse	*ChREBP*	Bricambert *et al.* [62]
	Mouse	*CYP8B1*	Pathak *et al.* [63]
	Mouse	*TNFα*, *CCL2*	Mikula *et al.* [64]
	Mouse	*PPARα* and related network genes	Jun *et al.* [65]
	Mouse	*ERO1α*, *LXRα*	Li *et al.* [33]
	Mouse	*SIRT1/macroH2A1.1*, *SIRT3*	Cao *et al.* [66]; Hirschey *et al.* [67]; Pazienza *et al.* [68]
	Human	NER	Schults *et al.* [69]

*MTTP* (microsomal triglyceride transfer protein), *Srebf2* (sterol regulatory element binding transcription factor 2), *Agpat3* (1-acylglycerol-3-phosphate oacyltransferase 3), *Esr1* (estrogen receptor 1), FASN (fatty acid synthase), *CDKN1a* (cyclin-dependent kinase inhibitor 1a), *PPARα* (peroxisome proliferator-activated receptors α), *INSIG* (insulin-induced gene), *PGC1α* (PPARγ coactivator 1α), *TFAM* (mitochondrial transcription factor A), *MT-ND6* (mitochondrially encoded NADH dehydrogenase 6), *PC* (pyruvate carboxylase), *ACLY* (ATP citrate lyase), *PLCG1* (phospholipase C-gamma-1), *IGF1* (insulin-like growth factor 1), *IGFBP2* (insulin-like growth factor binding protein 2), *PRKCE* (protein kinase C, epsilon), *GALNTL4* (putative polypeptide *N*-acetylgalactosaminyltransferase-like protein 4), *GRID1* (glutamate receptor δ-1 IP6K3 Inositol hexaphosphate kinase 3), *GPT2* (glutamic pyruvate transaminase 2), *DNAJA2* (DnaJ (Hsp40) homolog, subfamily A, member 2), *Rdh12* (retinol dehydrogenase 12), *Npas2* (neuronal PAS domain-containing protein 2), *ChREBP* (carbohydrate-responsive element-binding protein), *CYP8B1* (sterol 12α-hydroxylase), *TNFα* (tumor necrosis factor α), *CCL2* (chemokine C–C motif ligand 2), *ERO1α* (oxireductase endoplasmic reticulum oxidoreductin1α), *LXRα* (liver X receptor α), *SIRT1* (sirtuin 1), and NER (nucleotide excision repair).

## 3. Histone Modifications

Histone modifications mainly consist of acetylation, methylation, phosphorylation, and ubiquitylation. Among them, the histone acetylation patterns are the most heavily studied pattern. They are known to be regulated by histone acetyltransferases (HATs) and histone deacetylases (HDACs). To date, ample amounts of evidence have indicated changes in histone acetylation in NAFLD (Table 1, [61,62]). Studies in Japanese macaques have shown that a significant hyperacetylation of H3K14 in the fetal hepatic tissue was accompanied by upregulated acetylation at H3K9 and H3K18 [61]. A high-fat maternal diet was found to induce the depletion of HDAC1 protein in the fetal liver [61]. These findings indicate that HFD-induced maternal obesity can change fetal chromatin structure via histone modifications [61]. Carbohydrate-responsive element-binding protein (*ChREBP*) is a fundamental regulator in the progression of NAFLD. It acts as a transcriptional activator of lipogenic and glycolytic genes. It has been reported that HAT activator p300 and serine/threonine kinase salt-inducible kinase 2 (*SIK2*) are major upstream regulators of *ChREBP* activity. Glucose-activated p300 induced *ChREBP* hyperacetylation can promote its transcriptional activity. *SIK2* reduced *p300* HAT activity by phosphorylating Ser89. This attenuated *ChREBP*-mediated hepatic lipogenesis in mice. Both knockdown of *SIK2* and overexpression of *p300* exhibited led to hepatic steatosis and IR. The findings implicate that specific *SIK2* activators and *p300* inhibitors may be useful in pharmaceutical intervention of NAFLD [62]. Sterol 12α-hydroxylase (*CYP8B1*) is involved in cholic acid synthesis and cholesterol absorption. Retinoic acid-related orphan receptor α (*RORα*) recruited cAMP to induce histone acetylation of *CYP8B1* gene promoter. *RORα* is a major regulator of fasting induction and circadian rhythm of *CYP8B1*, which regulates bile acid synthesis and cholesterol levels. Antagonizing *RORα* activity might be a novel therapeutic strategy for the treatment of NAFLD and T2DM [63]. Tumor necrosis factor α (*TNFα*) and chemokine C-C motif ligand 2 (*CCL2*) are the important inflammatory mediators in the development of NAFLD. Chromatin immunoprecipitation assay showed an increase in histone H3 lysine 9 and 18 acetylation at *TNFα* and *CCL2* in mice with obesity [64]. These results suggest that high levels of expression of *TNFα* and *CCL2* at the chromatin level in fatty liver is associated with the alterations in histone H3 acetylation [64].

Data have shown that NAFLD is correlated with changes in the transcriptome caused by histone trimethylaton. Results have shown that hepatic lipid accumulation led to the aberrant histone H3K4 and H3K9 trimethylation in *PPARα* and lipid catabolism related genes, which increased the mRNA expression of these genes in HFD-fed mice. Results suggested that histone H3K4 an H3K9 trimethylation may contribute to hepatic steatosis and disease progression [65]. Lipogenesis and ER stress were promoted with the transgenerational alteration for oxireductase endoplasmic reticulum oxidoreductin1α (*ERO1α*), liver X receptor α (*LXRα*), histone methylations, and H3K9 histone methyltransferase. Low levels of accumulation of methylated histones in *ERO1-α* and *LXRα* gene promoters render the offsprings of HFD-induced mothers susceptible to hepatic steatosis and obesity [33].

Sirtuins (*SIRTs*) are part of the silent information regulator-2 family. Among the seven different sirtuins, *SIRT1* has been studied the most. Studies revealed that the deacetylation of *SIRT1* is responsible for the regulation of a variety of proteins that are involved in the pathophysiology of NAFLD [70]. *SIRT1* is involved in the regulation of glucose homeostasis, antihyperlipidemic activity, insulin sensitivity, oxidative stress, anti-inflammatory activity, and anti-aging activity [70]. Some studies have revealed a significant reduction in the expression of *SIRT1* in NAFLD animal models, and natural *SIRT1* activator showed protective effects on metabolic diseases [71]. Maternal HFD increased the fetal acetylation of histone H3K14 and decreased *SIRT1* expression in fetal livers. Maternal HFD altered the expression of downstream effector in NAFLD by regulating *SIRT1*. *SIRT1* maintains deacetylase activity on the peptide substrate of H3K14 acetylation *in vitro*. This activity may be abrogated due to the mutation of the catalytic domain of *SIRT1*. *SIRT1* modulates the fetal metabolome and epigenome as a molecular mediator under maternal HFD conditions [72]. Menin deficiency enhanced liver steatosis in HFD-fed mice. The adaptor menin recruits *SIRT1* to regulate *CD36* expression and triglyceride accumulation via histone deacetylation [66]. Conversely, *SIRT3* resides at the mitochondria and modulates fatty acid oxidation. HFD feeding results in hepatic mitochondrial protein hyperacetylation and decreases the *SIRT3* expression. *SIRT3* knockout mice developed hepatic steatosis and IR in the mice fed with HFD [67]. In this way, *SIRT1* and *SIRT3* are involved in the balance of metabolic and hepatic steatosis regulation through the epigenetic modification.

*MacroH2A1*, a target of *SIRT1*, is a variant of histone H2A and have two isoforms *macroH2A1.1* and *macroH2A1.2*. As an important transcriptional regulator, *macroH2A1* is involved in cell senescence and tumorigenic processes. The enhanced expression of *macroH2A1.1* reduced lipid deposition in hepatocytes, whereas *macroH2A1.2* was unable to do so. *MacroH2A1.1* ameliorated glucose metabolism and downregulated the expression of lipidogenic genes. Thus, *SIRT1/macroH2A1.1*-specific epigenetic regulation of lipid metabolism is associated with NAFLD development [68].

When NAFLD progressed to liver cancer, inflammation and oxidative stress played a critical role in the process. Inflammation activated neutrophil-mediated oxidative stress, which led to the reduced capacity of nucleotide excision repair (NER). Oxidative DNA damage further increased the risk of carcinogenesis in inflammatory tissues. Schults and colleagues proposed an attractive mechanism of the development of liver cancer in NAFLD patients. They observed that the neutrophilic influx with high expression of myeloperoxidase inhibited the damage recognition capacity, as indicated by staining for histone 2AX phosphorylation, which was paralleled by a reduction in NER capacity under hepatic inflammation conditions. Therefore, the reduction of NER capacity in the context of hepatic inflammation may have been caused by the reduced damage recognition [69].

## 4. MicroRNAs

MiRs are small, highly conserved noncoding RNAs of approximately 18–25 nucleotides in length that modulate translation and transcription of target genes. MiRs regulate a variety of biological functions in animals and human [73]. It appears that most miRs are repressed by epigenetic methylation of CpG islands [73]. MiRs perform a vital role in lipid metabolism and inflammation, some of which has been reported to be epigenetically regulated in NAFLD [74,75,76]. The miR alterations associated with NAFLD are summarized in Table 2. The miRs analysis showed a marked decrease in miR-122, miR-451, and miR-27 and increase in miR-200a, miR-200b, and miR-429 in rats fed HFD. These miRs are involved in the regulation of carbohydrate and lipid metabolism, signal transduction and apoptosis [77]. HFD-induced animal models showed considerable dysregulation of miRs and target genes. For instance, miR-467b expression was found to be markedly lower in the liver tissues of mice with hepatic steatosis than in those of otherwise normal mice. This difference was found to be inversely correlated with the expression of target gene of hepatic lipoprotein lipase (LPL). The interplay between miR-467b and LPL was associated with IR [78]. Decreased expression of miR-216 and miR-302a and increased expression of miR-24 were observed in the liver tissues with NAFLD, the target genes such as ATP-binding cassette transporter A1 (*ABCA1*), long-chain fatty acid elongation 6 (*ELOVL6*) and insulin-induced gene 1 (*Insig1*) are involved in hepatic fatty acid, cholesterol, and glucose metabolism [79,80]. However, some miRs levels (miR-705, miR-1224, miR-182, miR-183, miR-199a-3p, miR-200b, and miR-155) were upregulated in mice with methyl-deficient diet-induced NAFLD [81,82]. The polycomb group protein enhancer of zeste homolog 2 (*EZH2*) has been reported to regulate miRs by trimethylating Lys27 on histone H3. Inhibition of *EZH2* upregulated the inflammatory genes (*TNF-α* and *TGF-β*) and specific miRs (miR-200b and miR-155) and subsequently favored hepatic steatosis in NAFLD [83]. One study that focused on NASH patients showed 113 miRs expressed differentially. Among them, seven remained significant (miR-132, miR-150, miR-433, miR-28-3p, miR-511, miR-517a, and miR-671). The target genes of these miRs comprise obestatin gene, insulin receptor pathway genes, and inflammation-related genes. Additionally, miR-197 and miR-99 levels were correlated with liver fibrosis in NASH patients. MiRs from visceral adipose tissue (VAT) may be involved in the pathogenesis of NAFLD. These may serve as candidate biomarkers for NASH [84].

**Table 2:** MiRs alterations in NAFLD.

Study Subject	Upregulated MiRs	Downregulated MiRs	References
Human	miR-10b, miR-16, miR19a/b, miR-21, miR-27b-3p, miR-34a miR-122, miR125b, miR-192-5p, miR-451, miR-1290	miR-28-3p, miR-99a, miR-132, miR-146b, miR-150, miR-181d, miR-197, miR-296-5p, miR-433, miR-511, miR-517a, miR-671	Estep *et al.* [84], Tan *et al.* [85], Yamada *et al.* [86], Pirola *et al.* [87], Celikbilek *et al.* [88], Cermelli *et al.* [89], Clarke *et al.* [90], Miyaaki *et al.* [91], Min *et al.* [92], Zheng *et al.* [93], Cazanave *et al.* [94]
Mouse	miR-24, miR-33a, miR-34a, miR-122, miR-155, miR-181a, miR-182, miR-183, miR-192, miR-199a-3p/5p, miR-200b, miR-705, miR-1224	miR-92b-3p, miR-216, miR-302a, miR-328-3p, miR-467b, miR-484, miR-574-5p, miR-615-3p	Ahn *et al.* [78], Hoekstra *et al.* [79], Ng *et al.* [80], Dolganiuc *et al.* [81], Pogribny *et al.* [82], Tryndyak *et al.* [95], Derdak *et al.* [96], Li *et al.* [97], Li *et al.* [98], Miyamoto *et al.* [99]
Rat	miR-15b, miR-155, miR-200a/b, miR-429	miR-27, miR-122, miR-451	Alisi *et al.* [77], Vella *et al.* [83], Zhang *et al.* [100]

Although liver biopsy is still the gold standard of histopathological diagnosis of NAFLD, its invasive nature renders its performance difficult and unsatisfactory. A different technique, serum miR paneling, is a non-invasive diagnostic biomarker of NAFLD. The study showed the serum levels of miRs to be upregulated, including those of miR-122, miR-1290, miR-27b-3p, miR-192-5p, miR-21, miR-34a miR-15b miR-16, miR-451, miR19a/b, and miR125b [85,86,87,100]. Those of other miRs were lower in NAFLD patients. These included miR-146b, miR-99a, miR-181d, and miR-197 [88]. These miRs were involved in cell proliferation, glucose consumption, and TG storage. Moreover, the levels of serum miRs (miR-34a, miR-122, miR-181a, miR-192, and miR-200b) were strongly associated with inflammatory activity, fibrosis stage, and liver enzyme levels [89,95]. These findings suggest that the specific serum miRs may be used as noninvasive biomarkers of diagnosis and monitors of the severity of NAFLD.

MiR-122 serves as a key regulator of the metabolism of glucose and lipids in adult livers. The serum miR-122 levels remained high across the entire inductive time of NASH in mice, which was correlated with the extent of NASH [90]. Mostly, miR-122 circulates in argonaute 2-free forms and is expressed in the lipid-laden hepatocytes. MiR-122 increases alanine aminotransferase level by activating the translation at multiple sites of the coding gene [87]. In NAFLD patients, the hepatic miR-122 levels were lower in patients with mild steatosis than in those with severe steatosis. Conversely, patients with mild fibrosis showed higher levels of serum and hepatic miR-122 levels than patients with severe fibrosis. Consequently, the serum miR-122 level can be used as a predictive circulating marker of hepatic fibrosis in NAFLD patients [91].

MiR-34a, a transcriptional target of *p53*, is involved in the pathogenesis of NAFLD. *P53* inhibitor pifithrin-α p-nitro (*PFT*) abrogated the HFD-induced overexpressing of miR-34a and activate the *SIRT/PGC1α/PPARα* axis, which diminished hepatic TG deposition and ameliorated the liver steatosis [96]. NAFLD is also associated with free cholesterol and TG accumulation. NAFLD enhanced *SREBP-2* maturation, and reduced phosphorylation of HMG CoA reductase (HMGCR). MiR-34a overexpression repressed *SIRT1* with adenosine monophosphate-activated protein kinase (AMPK) and HMGCR dephosphorylation. HMGCR expression was related to free cholesterol, the histological extent of NAFLD and LDL-cholesterol. These findings indicate that the dysregulation of cholesterol metabolism may contribute to NAFLD and cardiovascular risks [92].

Based on studies, miRs participate in the regulation of some metabolism-related signal pathways. It has been proposed that cholesterol 7α-hydroxylase (*CYP7A1*)/steroid response element-binding protein 2 (*SREBP2*)/miR-33a axis performs a crucial role in the regulation of the synthesis of hepatic bile acid, cholesterol, and fatty acids. The overexpression of *CYP7A1* incerased *SREBP2*-related hepatic cholesterol synthesis and decreased hepatic fatty acid synthesis. Induction of SREBP2 subsequently activated miR-33a, which favored the lowered bile acid pool and elevated hepatic cholesterol levels in mice [97]. MiR-10b was found to be correlated with the steatosis level. *PPARα*, the direct target of miR-10b, showed markedly changed protein expression. The impact of miR-10b on the regulation of hepatic steatosis may offer a novel explanation for the pathophysiology of NAFLD [93]. The overexpression of miR199a-5p exacerbated fatty acid accumulation and repressed ATP levels and mtDNA content. Furthermore, miR199a-5p inhibited the expression of mitochondrial fatty acid β-oxidation-related genes via suppression of caveolin1 (CAV1) and PPARα signal pathway in obese mice and NAFLD patients. MiR199a-5p are involved in lipid metabolism and mitochondrial β-oxidation in NAFLD. MiR199a-5p overexpression may impair fatty acid β-oxidation and induce lipid deposition through the *CAV1* and *PPARα* pathway [98].

Some studies have demonstrated that miRs regulate lipoapoptosis and ER stress. Lipoapoptosis is associated with the upregulation of a pro-apoptotic protein p53-upregulated mediator of apoptosis (*PUMA*). Inhibition of miR-296-5p expression was observed in the livers of NASH patients compared with healthy controls. Moreover, miR-296-5p levels were inversely correlated with *PUMA* mRNA expression in liver specimens. The data suggest that miR-296-5p regulates *PUMA* expression during hepatic lipoapoptosis and increases in miR-296-5p expression may minimize apoptotic damage in NAFLD patients [94]. Palmitate is a crucial pathogenic event in NAFLD and activates the ER stress response through inducing the proapoptotic transcription factor *C/EBP* homologous protein (*CHOP*). Additionally, loss of miRs regulates lipoapoptosis under the ER stress conditions. Five miRs were downregulated under ER stress induced by palmitate in hepatocyte (miR-92b-3p, miR-328-3p, miR-484, miR-574-5p, and miR-615-3p). Enhancement of miR-615-3p levels with a precursor molecule decreased the *CHOP* expression and reduced the palmitate-induced hepatocyte apoptosis. These findings suggest elevation of miR-615-3p levels brings therapeutic benefit by suppressing palmitate-induced lipoapoptosis [99].

## 5. Potential Epigenetic Prevention or Therapy for NAFLD

Currently, NAFLD therapies are limited, and some studies have focused on identification of dietary natural compounds to provide new strategies for NAFLD [101]. It has been proven that dietary substances regulate fatty acid metabolism and are hypolipidemic agents [102]. Folate may affect expression of miRs related to the severity of NAFLD. Folate affects miRs expression, possibly through alterations in methylation levels in the genome [103]. The severity of NAFLD induced by folate-deficient diet is associated with the alteration of hepatic miRs expression, including miR-181a, miR-34a, miR-200b, and miR-221 [95]. Evidence has shown that the polyphenols decrease TG synthesis and oxidative stress in the liver, increase fatty acid oxidation, and reduce the risk of metabolic diseases by regulating specific miRs such as miR-122 [104]. Lycopene decreased the expression of miR-21 and its direct target fatty acid-binding protein 7 (*FABP7*) in mice fed stearic acid (SA), which blocked SA-evoked lipid accumulation. Lycopene may be used as a functional compound for NAFLD treatment [105]. Especially, grape seed proanthocyanidins not only promoted liver cholesterol efflux to produce HDL particles by imhibiting miR-33, but also repressed lipogenesis by decreasing miR-122. These agents showed hepatoprotective effects through their regulation of miRs’ expression associated with lipid metabolism [106].

Because it has been shown the overexpression of *SIRT1* will reduce the degree of hepatic steatosis in mice NAFLD models [72], activation of *SIRT1* with resveratrol, a natural *SIRT1* activator, protected against ER and IR stress and had positive effects on metabolic diseases, which is mediated by overexpression of oxygen-regulated protein 150. These findings indicated that *SIRT1* attenuated palmitate-induced ER and IR stress and the pharmacologic activation of *SIRT1* may offer a potential therapeutic strategy for NAFLD management [107]. It has been demonstrated that resveratrol reduced liver lipid deposition, upregulated acyl-coenzyme A oxydase (*ACO*) and carnitine palmitoyl transferase-Ia (*CPT-Ia*), downregulated acetyl-coenzyme A carboxylase (ACC) activity, which was mediated by AMPK/SIRT1 pathway activation [102]. Recently, it has been reported that hepatic steatosis could be ameliorated by drugs such as ursodeoxycholic acid (UDCA). UDCA repressed the miR-34a/SIRT1/p53 pathway in the liver of NAFLD rats. UDCA prevented miR-34a-dependent *SIRT1* suppression, *p53* acetylation, and apoptosis. Consistently, the overexpression of *p53* activated miR-34a/*SIRT1/p53*, which was in turn repressed by UDCA [108]. Conclusively, these potential modulators of NAFLD pathogenesis may provide new targets for therapeutic intervention.

## 6. Current Problems

Although the development of NAFLD is regulated by epigenetic alterations, including DNA methylation, histone modification and miRs, the investigation is just beginning and still faces many challenges. Clearly, various factors are involved in different stages of NAFLD, but the interactions among different factors and how they affect metabolistic homeostasis have not been clarified [109]. In addition, so far, few effective diagnostic and therapeutic targets exist. The possible difficulty results from the complicated biomolecular interactions and overlapping pathways in the setting of NAFLD [21]. It is important to exploit reliable biomolecules with high specificity and sensitivity based on the stage of NAFLD. Because epigenetic changes undergo transgenerational inheritance, animal models need to be developed to evaluate the influence of epigenetic modifications to the heritability of disease [110]. Nutritional cues regulated epigenetic alterations and affect the health in the offspring. Diet could change the metabolic gene expression and increase disease susceptibility. More studies should be undertaken to identify the optimal agents from diet for the prevention and intervention of NAFLD [37]. In recent years, the emergence of circulating miRs offers an option for using them as noninvasive diagnostic and therapeutic biomarkers. Nevertheless, some problems still hinder research, such as the identification of specific target miRs and the delivery mode, dosage, and period of treatment [111]. In addition, with regard to the observed potential biomolecules with hepatoprotective effect, further clinical trials are needed in order to evaluate the effect and safety of the management.

## 7. Conclusions and Perspectives

The accumulation of epigenetic evidence related to NAFLD has offered us a novel perspective into the pathogenesis of the disease and may contribute to identifying attractive diagnostic biomarkers and therapeutic strategies for NAFLD. Further research is needed to explore the role of epigenetics in the mechanism of hepatic steatosis and steatohepatitis, the interaction of environmental factors, and epigenetic modulation, in order to promote diagnostic and therapeutic approaches as well as reduce the morbidity and mortality in NAFLD.

## Abbreviations

NAFLD: non-alcoholic fatty liver disease
NASH: non-alcoholic steatohepatitis
HCC: hepatocellular carcinoma
TG: triglycerides
SS: simple steatosis
IR: insulin resistance
ER: endoplasmic reticulum
miRs: microRNAs
DNMTs: DNA methyltransferases
SAM: S-adenosylmethionine
HFD: high fat diet
MTTP: microsomal triglyceride transfer protein
HFS: high-fat-sucrose
Srebf2: sterol regulatory element binding transcription factor 2
Agpat3: 1-acylglycerol-3-phosphate Oacyltransferase 3
Esr1: estrogen receptor 1
FASN: fatty acid synthase
Cdkn1a: cyclin-dependent kinase inhibitor 1A
PPARs: Peroxisome proliferator-activated receptors
PGC1α: PPARγ coactivator 1α
TFAM: mitochondrial transcription factor A
HOMA-IR: homeostasis model assessment of IR
ROS: reactive oxygen species
MT-ND6: mitochondrially encoded NADH dehydrogenase 6
HATs: histone acetyltransferases
HDACs: histone deacetylases
ChREBP: carbohydrate-responsive element-binding protein
SIK2: serine/threonine kinase salt-inducible kinase 2
CYP8B1: sterol 12α-hydroxylase
RORα: retinoic acid-related orphan receptor α
TNFα: tumor necrosis factor α
CCL2: chemokine C-C motif ligand 2
ERO1α: endoplasmic reticulum oxidoreductin1α
LXRα: liver X receptor α
SIRTs: sirtuins
NER: nucleotide excision repair
LPL: lipoprotein lipase
Insig1: insulin-induced gene 1
ABCA1: ATP-binding cassette transporter A1
ELOVL6: long-chain fatty acid elongation 6
EZH2: enhancer of zeste homolog 2
VAT: visceral adipose tissue
PFT: P53 inhibitor pifithrin-α p-nitro
HMGCR: HMG CoA reductase
AMPK: adenosine monophosphate-activated protein kinase
CYP7A1: cholesterol 7α-hydroxylase
SREBP2: steroid response element-binding protein 2
CAV1: caveolin1
p53-upregulated mediator of apoptosis: PUMA
CHOP: C/EBP homologous protein
FABP7: fatty acid-binding protein 7
SA: stearic acid
ACO: acyl-coenzyme A oxydase
CPT-Ia: carnitine palmitoyl transferase-Ia
ACC: acetyl-coenzyme A carboxylase
UDCA: ursodeoxycholic acid

## References

[1] Marchesini G., Bugianesi E., Forlani G., Cerrelli F., Lenzi M., Manini R., Natale S., Vanni E., Villanova N., Melchionda N. (2003). Nonalcoholic fatty liver, steatohepatitis, and the metabolic syndrome. Hepatology.

[2] Younossi Z.M., Stepanova M., Afendy M., Fang Y., Younossi Y., Mir H., Srishord M. (2011). Changes in the prevalence of the most common causes of chronic liver diseases in the United States from 1988 to 2008. Clin. Gastroenterol. Hepatol..

[3] Dowman J.K., Tomlinson J.W., Newsome P.N. (2010). Pathogenesis of non-alcoholic fatty liver disease. QJM.

[4] Liu Q., Bengmark S., Qu S. (2010). The role of hepatic fat accumulation in pathogenesis of non-alcoholic fatty liver disease. Lipids Health Dis..

[5] Malik S.M., de Vera M.E., Fontes P., Shaikh O., Ahmad J. (2009). Outcome after liver transplantation for NASH cirrhosis. Am. J. Transplant..

[6] Siegel A.B., Zhu A.X. (2009). Metabolic syndrome and hepatocellular carcinoma. Cancer.

[7] Ong J.P., Pitts A., Younossi Z.M. (2008). Increased overall mortality and liver-related mortality in non-alcoholic fatty liver disease. J. Hepatol..

[8] Fan J.G. (2013). Epidemiology of alcoholic and nonalcoholic fatty liver disease in China. J. Gastroenterol. Hepatol..

[9] Amarapurkar D.N., Hashimoto E., Lesmana L.A., Sollano J.D., Chen P.J., Goh K.L. (2007). How common is non-alcoholic fatty liver disease in the Asia-Pacific region and are there local differences?. J. Gastroenterol. Hepatol..

[10] Zhou Y.J., Li Y.Y., Nie Y.Q., Huang C.M., Cao C.Y. (2012). Natural course of nonalcoholic fatty liver disease in southern China: A prospective cohort study. J. Dig. Dis..

[11] Wong V.W., Chu W.C., Wong G.L., Chan R.S., Chim A.M., Ong A., Yeung D.K., Yiu K.K., Chu S.H., Woo J. (2012). Prevalence of non-alcoholic fatty liver disease and advanced fibrosis in Hong Kong Chinese: A population study using proton-magnetic resonance spectroscopy and transient elastography. Gut.

[12] Adams L.A., Sanderson S., Lindor K.D., Angulo P. (2005). The histological course of nonalcoholic fatty liver disease: A longitudinal study of 103 patients with sequential liver biopsies. J. Hepatol..

[13] Fassio E., Alvarez E., Dominguez N., Landeira G., Longo C. (2004). Natural history of nonalcoholic steatohepatitis: A longitudinal study of repeat liver biopsies. Hepatology.

[14] Lindor K.D., Kowdley K.V., Heathcote E.J., Harrison M.E., Jorgensen R., Angulo P., Lymp J.F., Burgart L., Colin P. (2004). Ursodeoxycholic acid for treatment of nonalcoholic steatohepatitis: Results of a randomized trial. Hepatology.

[15] Ekstedt M., Franzén L.E., Mathiesen U.L., Thorelius L., Holmqvist M., Bodemar G., Kechagias S. (2006). Long-term follow-up of patients with NAFLD and elevated liver enzymes. Hepatology.

[16] Rafiq N., Bai C., Fang Y., Srishord M., McCullough A., Gramlich T. (2009). Long-term follow-up of patients with nonalcoholic fatty liver. Clin. Gastroenterol. Hepatol..

[17] Bechmann L.P., Hannivoort R.A., Gerken G., Hotamisligil G.S., Trauner M., Canbay A. (2012). The interaction of hepatic lipid and glucose metabolism in liver diseases. J. Hepatol..

[18] Haider D.G., Schindler K., Schaller G., Prager G., Wolzt M., Ludvik B. (2006). Increased plasma visfatin concentrations in morbidly obese subjects are reduced after gastric banding. J. Clin. Endocrinol. Metab..

[19] Farrell G.C., Larter C.Z. (2006). Nonalcoholic fatty liver disease: From steatosis to cirrhosis. Hepatology.

[20] Tacke F., Luedde T., Trautwein C. (2009). Inflammatory pathways in liver homeo-stasis and liver injury. Clin. Rev. Allergy Immunol..

[21] Berlanga A., Guiu-Jurado E., Porras J.A., Auguet T. (2014). Molecular pathways in non-alcoholic fatty liver disease. Clin. Exp. Gastroenterol..

[22] Neuschwander-Tetri B.A. (2010). Hepatic lipotoxicity and the pathogenesis of nonalcoholic steatohepatitis: The central role of nontriglyceride fatty acid metabolites. Hepatology.

[23] Lee J.H., Friso S., Choi S.W. (2014). Epigenetic mechanisms underlying the link between non-alcoholic fatty liver diseases and nutrition. Nutrients.

[24] Gu S.G., Pak J., Guang S., Maniar J.M., Kennedy S., Fire A. (2012). Amplification of siRNA in *Caenorhabditis elegans* generates a transgenerational sequence-targeted histone H3 lysine 9 methylation footprint. Nat. Genet..

[25] Heijmans B.T., Tobi E.W., Stein A.D., Putter H., Blauw G.J., Susser E.S., Slagboom P.E., Lumey L.H. (2008). Persistent epigenetic differences associated with prenatal exposure to famine in humans. Proc. Natl. Acad. Sci. USA.

[26] Hochberg Z., Feil R., Constancia M., Fraga M., Junien C., Carel J.C., Boileau P., Le Bouc Y., Deal C.L., Lillycrop K. (2011). Child health, developmental plasticity, and epigenetic programming. Endocr. Rev..

[27] Lomba A., Martinez J.A., Garcia-Diaz D.F., Paternain L., Marti A., Campion J., Milagro F.I. (2010). Weight gain induced by an isocaloric pair-fed high fat diet: A nutriepigenetic study on FASN and NDUFB6 gene promoters. Mol. Genet. Metab..

[28] Yoo C.B., Jones P.A. (2006). Epigenetic therapy of cancer: Past, present and future. Nat. Rev. Drug Discov..

[29] Paternain L., Garcia-Diaz D.F., Milagro F.I., Gonzalez-Muniesa P., Martinez J.A., Campion J. (2011). Regulation by chronic-mild stress of glucocorticoids, monocyte chemoattractant protein-1 and adiposity in rats fed on a high-fat diet. Physiol. Behav..

[30] Jiang M., Zhang Y., Liu M., Lan M.S., Fei J., Fan W., Gao X., Lu D. (2011). Hypermethylation of hepatic glucokinase and L-type pyruvate kinase promoters in high-fat diet-induced obese rats. Endocrinology.

[31] Sookoian S., Pirola C.J. (2012). DNA methylation and hepatic insulin resistance and steatosis. Curr. Opin. Clin. Nutr. Metab. Care.

[32] Pirola C.J., Gianotti T.F., Burgueño A.L., Rey-Funes M., Loidl C.F., Mallardi P., Martino J.S., Castaño G.O., Sookoian S. (2013). Epigenetic modification of liver mitochondrial DNA is associated with histological severity of nonalcoholic fatty liver disease. Gut.

[33] Li J., Huang J., Li J.S., Chen H., Huang K., Zheng L. (2012). Accumulation of endoplasmic reticulum stress and lipogenesis in the liver through generational effects of high fat diets. J. Hepatol..

[34] Tian Y., Wong V.W., Chan H.L., Cheng A.S. (2013). Epigenetic regulation of hepatocellular carcinoma in non-alcoholic fatty liver disease. Semin. Cancer Biol..

[35] Dawson M.A., Kouzarides T. (2012). Cancer epigenetics: From mechanism to therapy. Cell.

[36] Bruce K.D., Cagampang F.R. (2011). Epigenetic priming of the metabolic syndrome. Toxicol. Mech. Methods.

[37] Iacobazzi V., Castegna A., Infantino V., Andria G. (2013). Mitochondrial DNA methylation as a next-generation biomarker and diagnostic tool. Mol. Genet. Metab..

[38] Wang L.J., Zhang H.W., Zhou J.Y., Liu Y., Yang Y., Chen X.L., Zhu C.H., Zheng R.D., Ling W.H., Zhu H.L. (2014). Betaine attenuates hepatic steatosis by reducing methylation of the MTTP promoter and elevating genomic methylation in mice fed a high-fat diet. J. Nutr. Biochem..

[39] Li Y.Y. (2012). Genetic and epigenetic variants influencing the development of nonalcoholic fatty liver disease. World J. Gastroenterol..

[40] Kalhan S.C., Edmison J., Marczewski S., Dasarathy S., Gruca L.L., Bennett C., Duenas C., Lopez R. (2011). Methionine and protein metabolism in non-alcoholic steatohepatitis: Evidence for lower rate of transmethylation of methionine. Clin. Sci. (Lond.).

[41] Niculescu M.D., Zeisel S.H. (2002). Diet, methyl donors and DNA methylation: Interactions between dietary folate, methionine and choline. J. Nutr..

[42] Zivkovic A.M., Bruce German J., Esfandiari F., Halsted C.H. (2009). Quantitative lipid metabolomic changes in alcoholic micropigs with fatty liver disease. Alcohol. Clin. Exp. Res..

[43] Da Silva R.P., Kelly K.B., Al Rajabi A., Jacobs R.L. (2014). Novel insights on interactions between folate and lipid metabolism. Biofactors.

[44] Chang X., Yan H., Fei J., Jiang M., Zhu H., Lu D., Gao X. (2010). Berberine reduces methylation of the MTTP promoter and alleviates fatty liver induced by a high-fat diet in rats. J. Lipid Res..

[45] Cordero P., Campion J., Milagro F.I., Martinez J.A. (2013). Transcriptomic and epigenetic changes in early liver steatosis associated to obesity: Effect of dietary methyl donor supplementation. Mol. Genet. Metab..

[46] Cordero P., Gomez-Uriz A.M., Campion J., Milagro F.I., Martinez J.A. (2013). Dietary supplementation with methyl donors reduces fatty liver and modifies the fatty acid synthase DNA methylation profile in rats fed an obesogenic diet. Genes Nutr..

[47] Tryndyak V.P., Han T., Muskhelishvili L., Fuscoe J.C., Ross S.A., Beland F.A., Pogribny I.P. (2011). Coupling global methylation and gene expression profiles reveal key pathophysiological events in liver injury induced by a methyl-deficient diet. Mol. Nutr. Food Res..

[48] Wolff G.L., Kodell R.L., Moore S.R., Cooney C.A. (1998). Maternal epigenetics and methyl supplements affect agouti gene expression in Avy/a mice. FASEB J..

[49] Dudley K.J., Sloboda D.M., Connor K.L., Beltrand J., Vickers M.H. (2011). Offspring of mothers fed a high fat diet display hepatic cell cycle inhibition and associated changes in gene expression and DNA methylation. PLoS One.

[50] Pruis M.G., Lendvai A., Bloks V.W., Zwier M.V., Baller J.F., de Bruin A., Groen A.K., Plösch T. (2014). Maternal western diet primes non-alcoholic fatty liver disease in adult mouse offspring. Acta Physiol. (Oxf.).

[51] Tiao M.M., Huang L.T., Chen C.J., Sheen J.M., Tain Y.L., Chen C.C., Kuo H.C., Huang Y.H., Tang K.S., Chu E.W. (2014). Melatonin in the regulation of liver steatosis following prenatal glucocorticoid exposure. Biomed. Res. Int..

[52] Gavrilova O., Haluzik M., Matsusue K., Cutson J.J., Johnson L., Dietz K.R., Nicol C.J., Vinson C., Gonzalez F.J., Reitman M.L. (2003). Liver peroxisome proliferator-activated receptor gamma contributes to hepatic steatosis, triglyceride clearance, and regulation of body fat mass. J. Biol. Chem..

[53] Tyagi S., Gupta P., Saini A.S., Kaushal C., Sharma S. (2011). The peroxisome proliferator-activated receptor: A family of nuclear receptors role in various diseases. J. Adv. Pharm. Technol. Res..

[54] Sie K.K., Li J., Ly A., Sohn K.J., Croxford R., Kim Y.I. (2013). Effect of maternal and postweaning folic acid supplementation on global and gene-specific DNA methylation in the liver of the rat offspring. Mol. Nutr. Food Res..

[55] Sookoian S., Rosselli M.S., Gemma C., Burgueño A.L., Fernández Gianotti T., Castaño G.O., Pirola C.J. (2010). Epigenetic regulation of insulin resistance in nonalcoholic fatty liver disease: Impact of liver methylation of the peroxisome proliferator-activated receptor γ coactivator 1α promoter. Hepatology.

[56] Chen G., Broséus J., Hergalant S., Donnart A., Chevalier C., Bolaños-Jiménez F., Guéant J.L., Houlgatte R. (2015). Identification of master genes involved in liver key functions through transcriptomics and epigenomics of methyl donor deficiency in rat: Relevance to nonalcoholic liver disease. Mol. Nutr. Food Res..

[57] Carabelli J., Burgueno A.L., Rosselli M.S., Gianotti T.F., Lago N.R., Pirola C.J., Sookoian S. (2011). High fat diet-induced liver steatosis promotes an increase in liver mitochondrial biogenesis in response to hypoxia. J. Cell. Mol. Med..

[58] Turrens J.F. (2003). Mitochondrial formation of reactive oxygen species. J. Physiol..

[59] Murphy S.K., Yang H., Moylan C.A., Pang H., Dellinger A., Abdelmalek M.F., Garrett M.E., Ashley-Koch A., Suzuki A., Tillmann H.L. (2013). Relationship between methylome and transcriptome in patients with nonalcoholic fatty liver disease. Gastroenterology.

[60] Ahrens M., Ammerpohl O., von Schönfels W., Kolarova J., Bens S., Itzel T., Teufel A., Herrmann A., Brosch M., Hinrichsen H. (2013). DNA methylation analysis in nonalcoholic fatty liver disease suggests distinct disease-specific and remodeling signatures after bariatric surgery. Cell Metab..

[61] Aagaard-Tillery K.M., Grove K., Bishop J., Ke X., Fu Q., McKnight R., Lane R.H. (2008). Developmental origins of disease and determinants of chromatin structure: Maternal diet modifies the primate fetal epigenome. J. Mol. Endocrinol..

[62] Bricambert J., Miranda J., Benhamed F., Girard J., Postic C., Dentin R. (2010). Salt-inducible kinase 2 links transcriptional coactivator P300 phosphorylation to the prevention of ChREBP-dependent hepatic steatosis in mice. J. Clin. Investig..

[63] Pathak P., Li T., Chiang J.Y. (2013). Retinoic acid-related orphan receptor α regulates diurnal rhythm and fasting induction of sterol 12α-hydroxylase in bile acid synthesis. J. Biol. Chem..

[64] Mikula M., Majewska A., Ledwon J.K., Dzwonek A., Ostrowski J. (2014). Obesity increases histone H3 lysine 9 and 18 acetylation at TNFα and CCL2 genes in mouse liver. Int. J. Mol. Med..

[65] Jun H.J., Kim J., Hoang M.H., Lee S.J. (2012). Hepatic lipid accumulation alters global histone H3 lysine 9 and 4 trimethylation in the peroxisome proliferator-activated receptor α network. PLoS One.

[66] Cao Y., Xue Y., Xue L., Jiang X., Wang X., Zhang Z., Yang J., Lu J., Zhang C., Wang W. (2013). Hepatic menin recruits SIRT1 to control liver steatosis through histone deacetylation. J. Hepatol..

[67] Hirschey M.D., Shimazu T., Jing E., Grueter C.A., Collins A.M., Aouizerat B., Stančáková A., Goetzman E., Lam M.M., Schwer B. (2011). SIRT3 deficiency and mitochondrial protein hyperacetylation accelerate the development of the metabolic syndrome. Mol. Cell.

[68] Pazienza V., Borghesan M., Mazza T., Sheedfar F., Panebianco C., Williams R., Mazzoccoli G., Andriulli A., Nakanishi T., Vinciguerra M. (2014). SIRT1-metabolite binding histone macroH2A1.1 protects hepatocytes against lipid accumulation. Aging (Albany NY).

[69] Schults M.A., Nagle P.W., Rensen S.S., Godschalk R.W., Munnia A., Peluso M., Claessen S.M., Greve J.W., Driessen A., Verdam F.J. (2012). Decreased nucleotide excision repair in steatotic livers associates with myeloperoxidase-immunoreactivity. Mutat. Res..

[70] Colak Y., Yesil A., Mutlu H.H., Caklili O.T., Ulasoglu C., Senates E., Takir M., Kostek O., Yilmaz Y., Enc F.Y. (2014). A potential treatment of non-alcoholic fatty liver disease with SIRT1 activators. J. Gastrointest. Liver Dis..

[71] Colak Y., Ozturk O., Senates E., Tuncer I., Yorulmaz E., Adali G., Doganay L., Enc F.Y. (2011). SIRT1 as a potential therapeutic target for treatment of nonalcoholic fatty liver disease. Med. Sci. Monit..

[72] Suter M.A., Chen A., Burdine M.S., Choudhury M., Harris R.A., Lane R.H., Friedman J.E., Grove K.L., Tackett A.J., Aagaard K.M. (2012). A maternal high-fat diet modulates fetal SIRT1 histone and protein deacetylase activity in nonhuman primates. FASEB J..

[73] Wang Z., Yao H., Lin S., Zhu X., Shen Z., Lu G., Poon W.S., Xie D., Lin M.C., Kung H.F. (2013). Transcriptional and epigenetic regulation of human microRNAs. Cancer Lett..

[74] Finch M.L., Marquardt J.U., Yeoh G.C., Callus B.A. (2014). Regulation of microRNAs and their role in liver development, regeneration and disease. Int. J. Biochem. Cell. Biol..

[75] Ferreira D.M., Simão A.L., Rodrigues C.M., Castro R.E. (2014). Revisiting the metabolic syndrome and paving the way for microRNAs in non-alcoholic fatty liver disease. FEBS J..

[76] Panera N., Gnani D., Crudele A., Ceccarelli S., Nobili V., Alisi A. (2014). MicroRNAs as controlled systems and controllers in non-alcoholic fatty liver disease. World J. Gastroenterol..

[77] Alisi A., Da Sacco L., Bruscalupi G., Piemonte F., Panera N., de Vito R., Leoni S., Bottazzo G.F., Masotti A., Nobili V. (2011). Mirnome analysis reveals novel molecular determinants in the pathogenesis of diet-induced nonalcoholic fatty liver disease. Lab. Investig..

[78] Ahn J., Lee H., Chung C.H., Ha T. (2011). High fat diet induced downregulation of microRNA-467b increased lipoprotein lipase in hepatic steatosis. Biochem. Biophys. Res. Commun..

[79] Hoekstra M., van der Sluis R.J., Kuiper J., van Berkel T.J. (2012). Nonalcoholic fatty liver disease is associated with an altered hepatocyte microRNA profile in LDL receptor knockout mice. J. Nutr. Biochem..

[80] Ng R., Wu H., Xiao H., Chen X., Willenbring H., Steer C.J., Song G. (2014). Inhibition of microRNA-24 expression in liver prevents hepatic lipid accumulation and hyperlipidemia. Hepatology.

[81] Dolganiuc A., Petrasek J., Kodys K., Catalano D., Mandrekar P., Velayudham A., Szabo G. (2009). MicroRNA expression profile in Lieber-DeCarli diet-induced alcoholic and methionine choline deficient diet-induced nonalcoholic steatohepatitis models in mice. Alcohol. Clin. Exp. Res..

[82] Pogribny I.P., Starlard-Davenport A., Tryndyak V.P., Han T., Ross S.A., Rusyn I., Beland F.A. (2010). Difference in expression of hepatic microRNAs miR-29c, miR-34a, miR-155, and miR-200b is associated with strain-specific susceptibility to dietary nonalcoholic steatohepatitis in mice. Lab. Investig..

[83] Vella S., Gnani D., Crudele A., Ceccarelli S., de Stefanis C., Gaspari S., Nobili V., Locatelli F., Marquez V.E., Rota R. (2013). EZH2 down-regulation exacerbates lipid accumulation and inflammation in *in vitro* and *in vivo* NAFLD. Int. J. Mol. Sci..

[84] Estep M., Armistead D., Hossain N., Elarainy H., Goodman Z., Baranova A., Chandhoke V., Younossi Z.M. (2010). Differential expression of miRNAs in the visceral adipose tissue of patients with non-alcoholic fatty liver disease. Aliment Pharmacol. Ther..

[85] Tan Y., Ge G., Pan T., Wen D., Gan J. (2014). A pilot study of serum microRNAs panel as potential biomarkers for diagnosis of nonalcoholic fatty liver disease. PLoS One.

[86] Yamada H., Suzuki K., Ichino N., Ando Y., Sawada A., Osakabe K., Sugimoto K., Ohashi K., Teradaira R., Inoue T. (2013). Associations between circulating microRNAs (miR-21, miR-34a, miR-122 and miR-451) and non-alcoholic fatty liver. Clin. Chim. Acta.

[87] Pirola C.J., Gianotti T.F., Castaño G.O., Mallardi P., Martino J.S., Gonzalez Lopez Ledesma M.M., Flichman D., Mirshahi F., Sanyal A.J., Sookoian S. (2014). Circulating microRNA signature in non-alcoholic fatty liver disease: From serum non-coding RNAs to liver histology and disease pathogenesis. Gut.

[88] Celikbilek M., Baskol M., Taheri S., Deniz K., Dogan S., Zararsiz G., Gursoy S., Guven K., Ozbakır O., Dundar M. (2014). Circulating microRNAs in patients with non-alcoholic fatty liver disease. World J. Hepatol..

[89] Cermelli S., Ruggieri A., Marrero J.A., Ioannou G.N., Beretta L. (2011). Circulating microRNAs in patients with chronic hepatitis C and non-alcoholic fatty liver disease. PLoS One.

[90] Clarke J.D., Sharapova T., Lake A.D., Blomme E., Maher J., Cherrington N.J. (2014). Circulating microRNA 122 in the methionine and choline-deficient mouse model of non-alcoholic steatohepatitis. J. Appl. Toxicol..

[91] Miyaaki H., Ichikawa T., Kamo Y., Taura N., Honda T., Shibata H., Milazzo M., Fornari F., Gramantieri L., Bolondi L. (2014). Significance of serum and hepatic microRNA-122 levels in patients with non-alcoholic fatty liver disease. Liver Int..

[92] Min H.K., Kapoor A., Fuchs M., Mirshahi F., Zhou H., Maher J., Kellum J., Warnick R., Contos M.J., Sanyal A.J. (2012). Increased hepatic synthesis and dysregulation of cholesterol metabolism is associated with the severity of nonalcoholic fatty liver disease. Cell Metab..

[93] Zheng L., Lv G.C., Sheng J., Yang Y.D. (2010). Effect of miRNA-10b in regulating cellular steatosis level by targeting PPAR-α expression, a novel mechanism for the pathogenesis of NAFLD. J. Gastroenterol. Hepatol..

[94] Cazanave S.C., Mott J.L., Elmi N.A., Bronk S.F., Masuoka H.C., Charlton M.R., Gores G.J. (2011). A role for miR-296 in the regulation of lipoapoptosis by targeting PUMA. J. Lipid Res..

[95] Tryndyak V.P., Latendresse J.R., Montgomery B., Ross S.A., Beland F.A., Rusyn I., Pogribny I.P. (2012). Plasma microRNAs are sensitive indicators of inter-strain differences in the severity of liver injury induced in mice by a choline- and folate-deficient diet. Toxicol. Appl. Pharmacol..

[96] Derdak Z., Villegas K.A., Harb R., Wu A.M., Sousa A., Wands J.R. (2013). Inhibition of p53 attenuates steatosis and liver injury in a mouse model of non-alcoholic fatty liver disease. J. Hepatol..

[97] Li T., Francl J.M., Boehme S., Chiang J.Y. (2013). Regulation of cholesterol and bile acid homeostasis by the cholesterol 7α-hydroxylase/steroid response element-binding protein 2/microRNA-33a axis in mice. Hepatology.

[98] Li B., Zhang Z., Zhang H., Quan K., Lu Y., Cai D., Ning G. (2014). Aberrant miR199a-5p/caveolin1/PPARα axis in hepatic steatosis. J. Mol. Endocrinol..

[99] Miyamoto Y., Mauer A.S., Kumar S., Mott J.L., Malhi H. (2014). Mmu-miR-615–3p regulates lipoapoptosis by inhibiting C/EBP homologous protein. PLoS One.

[100] Zhang Y., Cheng X., Lu Z., Wang J., Chen H., Fan W., Gao X., Lu D. (2013). Upregulation of miR-15b in NAFLD models and in the serum of patients with fatty liver disease. Diabetes Res. Clin. Pract..

[101] Pan M.H., Lai C.S., Tsai M.L., Ho C.T. (2014). Chemoprevention of nonalcoholic fatty liver disease by dietary natural compounds. Mol. Nutr. Food Res..

[102] Alberdi G., Rodriguez V.M., Macarulla M.T., Miranda J., Churruca I., Portillo M.P. (2013). Hepatic lipid metabolic pathways modified by resveratrol in rats fed an obesogenic diet. Nutrition.

[103] Iizuka K., Horikawa Y. (2008). ChREBP: A glucose-activated transcription factor involved in the development of metabolic syndrome. Endocr. J..

[104] Bladé C., Baselga-Escudero L., Salvadó M.J., Arola-Arnal A. (2013). miRNAs, polyphenols, and chronic disease. Mol. Nutr. Food Res..

[105] Ahn J., Lee H., Jung C.H., Ha T. (2012). Lycopene inhibits hepatic steatosis via microRNA-21-induced downregulation of fatty acid-binding protein 7 in mice fed a high-fat diet. Mol. Nutr. Food Res..

[106] Baselga-Escudero L., Blade C., Ribas-Latre A., Casanova E., Salvado M.J., Arola L., Arola-Arnal A. (2012). Grape seed proanthocyanidins repress the hepatic lipid regulators miR-33 and miR-122 in rats. Mol. Nutr. Food Res..

[107] Jung T.W., Lee K.T., Lee M.W., Ka K.H. (2012). SIRT1 attenuates palmitate-induced endoplasmic reticulum stress and insulin resistance in HepG2 cells via induction of oxygen-regulated protein 150. Biochem. Biophys. Res. Commun..

[108] Castro R.E., Ferreira D.M., Afonso M,B., Borralho P.M., Machado M.V., Cortez-Pinto H., Rodrigues C.M. (2013). miR-34a/SIRT1/p53 is suppressed by ursodeoxycholic acid in the rat liver and activated by disease severity in human non-alcoholic fatty liver disease. J. Hepatol..

[109] Gori M., Arciello M., Balsano C. (2014). MicroRNAs in nonalcoholic fatty liver disease: Novel biomarkers and prognostic tools during the transition from steatosis to hepatocarcinoma. Biomed. Res. Int..

[110] Zimmer V., Lammert F. (2011). Genetics and epigenetics in the fibrogenic evolution of chronic liver diseases. Best Pract. Res. Clin. Gastroenterol..

[111] Moore K.J., Rayner K.J., Suárez Y., Fernández-Hernando C. (2010). microRNAs and cholesterol metabolism. Trends Endocrinol. Metab..

